# Tribological and Structural Properties of Copper-Coated 3D-Printed Parts from Biodegradable Polymers

**DOI:** 10.3390/mi16010100

**Published:** 2025-01-16

**Authors:** Mihaela Feraru (Ilie), Simona-Nicoleta Mazurchevici, Nicoleta-Monica Lohan, Marcelin Benchea, Fabian Cezar Lupu, Dumitru Nedelcu

**Affiliations:** 1 Department of Machine Manufacturing Technology, “Gheorghe Asachi” Technical University of Iasi, 700050 Iasi, Romania; iliemiha2005@gmail.com; 2 Faculty of Materials Science and Engineering, “Gheorghe Asachi” Technical University of Iasi, Blvd. Dimitrie Mangeron 71A, 700050 Iasi, Romania; nicoleta-monica.lohan@academic.tuiasi.ro; 3 Faculty of Mechanics, “Gheorghe Asachi” Technical University of Iasi, Str. Prof. Dr. Doc. Dimitrie Mangeron, No. 43, 700050 Iasi, Romania; marcelin.benchea@academic.tuiasi.ro (M.B.); fabian-cezar.lupu@academic.tuiasi.ro (F.C.L.); 4 Mechanical Engineering Department, Technical Sciences Academy of Romania, 030167 Bucharest, Romania

**Keywords:** copper coating, biodegradable plastic, structure, thermal behavior, tribology

## Abstract

This manuscript highlights the behavior of biodegradable polymers (PLA and HD PLA Green) coated with two distinct bronze alloy powders, Metco 51F-NS (Cu 9.5Al 1.2Fe) and Metco 445 (Cu 9.5Al). The coating was realized on printed samples by using the Atmospheric Plasma Spray (APS) technique. The current investigation will explain the results related to the surface quality, micro-structure, morphology, and thermal and tribological properties. Thus, from a structural point of view, the most uniform deposition was obtained in the case of composite powder Metco 51F-NS. The thermal behavior of the samples coated with copper-based powder achieved stability up to temperatures slightly above 200 °C, with carbonization of the matrix structure taking place around 350 °C. The micro-indentation and scratch analysis responses were significantly influenced by the semicrystalline structure of the samples and the presence of the powder compounds. Based on the increased characteristics of the coated samples, the authors of the present paper consider that parts made of biodegradable polymers and coated with copper microparticles are appropriate for some applications which take place in adverse operating conditions.

## 1. Introduction

In addition to protecting 3D-printed objects from external factors, coatings applied to their surfaces also enhance their esthetically pleasing qualities. Understanding the chemical and physical characteristics of surfaces made of polymers is crucial for developing an ideal surface system [1,2]. The surface treatment of polymers can only be accomplished with the right coatings because of their unique surface features. In fact, several resins and solvents such as polyurethane, polyester, and acrylics are typically found in such coatings [3,4]. The coatings’ types and qualities, as well as the 3D-printed objects’ shapes and sizes, determine the technologies that can be used to apply them [5]. The excessive surface roughness of 3D-printed items is another major concern with layer-by-layer fabrication. Several pre- and post-processing techniques, such as plasma treatment, chemical treatments, laser micro-machining, and sanding, can be used to control and enhance the surface finishing of manufactured items [6,7].

There are reports in the scientific literature on surface treatment of 3D-printed items that were generated with different raw materials and coated with various materials and procedures. What follows is a synopsis of the results presented in a number of articles that were chosen because of their relevance to the current investigation. A technique that has grown in popularity in recent years, thermal spraying (TS), enables the application of nearly any material to nearly any substrate [8,9,10]. It is possible to melt the powdered material using electrical, chemical, or kinetic heat, depending on the process. All of these methods rely on a gun to melt and accelerate the coating precursor or raw material towards the target using either an electric arc, flame, plasma, or warmed gas. The coating precursor is a jet of powdered metal, plastic, ceramic, wire, or rod that, upon impact, sticks to the target. Coating layers are formed when incoming particles adhere to one another and cover the target entirely [10,11,12].

A classification of the thermal sputter coating processes according to the type of energy source used during deposition [13,14], can be the following: energy from liquid melt (liquid sputtering); energy from gas combustion (flame, detonation, and high velocity sputtering; kinetic energy (kinetic cold sputtering); energy from electric discharge (plasma sputtering and arc sputtering); and laser beam energy (laser sputtering/plating). Atmospheric Plasma Spraying (APS) is part of TS technology [15]. The main areas of use of this technology are closely related to the improvement of the surface characteristics of the substrate: wear resistance (abrasion, erosion, and friction); resistance to corrosion and oxidation; electrical conductivity or insulation; thermal or magnetic conductivity; biocompatibility; etc. [16].

The key benefits of APS thermal coating are increased part/mark lifetime, decreased expenses, reduced downtime/maintenance, improved productivity, and improved product quality via improving functional qualities. The methods’ drawbacks include high working temperatures that can degrade the substrate and rapid powder cooling due to the cold substrate. Selecting the right spraying distance and duration can mitigate these drawbacks. Preheating the substrate reduces the effect of rapid particle cooling [13]. The main classes of materials used for the deposition of layers using the APS method are as follows: metallic micro/nanoparticles such as (gold, silver, copper, aluminum, cobalt, zinc, cadmium, etc.) [17]; micro/nanoparticles based on metal oxides such as iron oxide, aluminum oxides, silicon, zinc, titanium, and cerium [18]; micro/nanoparticles based on ceramic such as silica (SiO_2_), alumina (Al_2_O_3_), zirconium (ZrO_2_), titanium dioxide (TiO_2_), and silicon carbide (SiC) [19,20]. However, customers are becoming more aware of the environmental impact of petrochemical polymers [21,22,23]. Additionally, a global trend away from polymers with hundreds of years of durability has refocused attention on formerly inaccessible regions [24,25]. Over the past decade, bio-based polymer applications, technology, and case studies have altered drastically [26,27,28,29,30]. New biopolymers including PLA, PHA, and bio-based drop-in PET and PE/PP are the most important [31]. The current analysis predicts the greatest market growth for bio-based PET and PLA [32]. The substrate type, deposition surface quality, powder type, gas type, and deposition process parameters all determine the coating deposition rate. The wide range of mechanical and thermal properties of polymers and composites determines their reactivity to particles, requiring exact adaption of all these aspects to achieve the best indicators after coating. Because of its low hardness, fatigue resistance, erosion resistance, and great elasticity, only soft metal powder may be directly coated on polymers [33,34]. Temperature and gas pressure mainly affect powder velocity, kinetic energy, strain rate, impact temperature, and spray-ability under these conditions [35,36,37,38,39,40,41,42]. The number of passes, spray distance, and gun speed are secondary characteristics [43,44,45,46,47]. These settings can adjust particle impact energy, deposition spot size, and substrate thermal softening under the hot gas jet [44]. These physical considerations greatly affect coating properties [13]. In this respect, researchers have investigated over the last few years the possibility to improve the surface characteristics of polymeric materials by depositing copper layers on polymeric substrates. The CS (cold spray) coating method was used in studies such as Lupoi and O’Neill [44] who cold-sprayed 50–100 μm powders of Cu, Al, and Sn onto various polymers. They claimed that low density prevented substrate degradation and encouraged powder deposition. Mixing powder with tougher metal or ceramic particles improves spray-ability. The harder particles in the combination roughen the substrate and promote particle interlocking. They also micro-grind the coating, compacting the layers and reducing porosity; Chu et al. [48] increased the efficiency of layer deposition by 3–6 times through mixing Sn powder with 10% Cu, Zn, or Al powder to fix the erosion. The deposition was realized on a CFRP substrate. The efficiency of layer deposition enhancement is thermodynamically caused by the secondary (hard) particles’ kinetic energy being converted into strain energy and the primary (soft) particles being heated. The powdered second ingredient helps melt the first one; Bortolussi et al. [49] have studied CFRP coated with Cu and polymer powder. They found that the polymer powder in the deposition mixture can bind the metal powder while retaining a percolated network of metal particles for macroscopic conductivity. By selecting sputtering conditions, powders with specified forms, sizes, and flow ratios can create micro-structures with distinct electrical and bonding capabilities; mixing powders customize particle fluidity. Sn sputtered at 325 °C quickly clogged the nozzle [50]. However, combining Sn powder with 30% Cu prevented clogging at gas temperatures < 350◦C, perhaps due to Cu particles acting as “nozzle sweepers” [51]. Combining soft metal powder and ceramic particles reduces nozzle blockage [52]. Rokni et al. examined particle hardness effects [53]. Researchers found that local softening in thermoplastics makes cold spraying metals on them more effective than on thermosets [54,55,56]. Małachowska et al. [57] employed low-pressure cold spray to metallize polymers and measure the adhesion strength of the coatings. R. N. Raoelison [58] compares a copper/PEEK interface generated by high-pressure cold spraying to a copper/copper combination to study material dissymmetry and bond strength.

Concerning thermal spraying and the APS method in particular, studies were also performed, such as in the case of carbon fiber reinforced polymers where the use of the APS method for the deposition of a copper layer was investigated by crack propagation tracking tests and interlaminar breakage tests. These methods have provided valuable information on the mechanical behavior of the deposited layer and its interaction with the substrate, the results being documented in the scientific literature [59]. Also, concerning CFRP substrates, research has focused on the determination of the adhesion strength of the deposited copper layer by APS. These studies have highlighted the performance characteristics of the deposited layer under specific conditions, data of which are included in the reference [59]. For carbon fiber-reinforced epoxy, extensive analyses of the properties of the deposited layer were performed. The studies included X-ray photoelectron spectroscopy (XPS) determinations, evaluations of the percentage of bonds created, topography analysis of the treated and untreated samples (by thermal, chemical, and mechanical methods), contact angle measurements, and surface energy determination. In addition, scanning electron microscopy (SEM) provided detailed images of the surface to evaluate the structure and homogeneity of the deposited layer. These analyses allowed a thorough understanding of the adhesion process, as reported in reference [60]. The results of these studies contribute significantly to the understanding of adhesion processes and to the optimization of coating technologies for composite materials used in industries such as aeronautics, automotive, or electronics. There is still a lack of publications dealing with the properties of 3D-printed products coated with microparticles based on copper. The aim of the present research was to assess the properties of two different biodegradable substrates (PLA and HD PLA Green). The substrates were coated with two different copper-based coatings, Metco 51F-NS and Metco 445. The hypothesis was that surface physical, morphological, and tribological properties of 3D-printed products depend on substrate type and the type of applied coating. It was additionally assumed that adhesion strength between the substrate and coating depends on the type of both surface system components. In order to determine good adhesion between the selected substrates and coatings, morphological, structural, tribological, and thermal analyses were carried out in order to make recommendations for their industrial applications.

## 2. Materials and Methods

Institute PLA filament (Raise3D company, Irvine, CA, USA) and HD PLA Green (Fiberlogy company, Brzezie, Polonia) were used for printing the samples (to be coated) by using a FDM printer—Raise3D Pro2Plus 3D (Sintratec, Brugg, Switzerland). The printing parameters were as follows: orientation of the sample on the printing table—on the edge; layer thickness 0.1 mm; deposition speed 80 mm/min. In what concerns the parallelepipedic samples, dimensions were as follows: length—70 mm; width—48 mm; thickness—10 mm.

Two powders (purchased from the manufacturer Oerlikon Metco—Bella Vista (New South Wales, Australia), with the following trade names and chemical composition and features, were utilized for the coating [61]:

- Metco 51F-NS (Bronze Alloy—Cu 9.5Al 1.2Fe—nominal composition; chemical composition wt% nominal: 8.5–10.75Al, 0.5–2.0Fe, max 0.5 other)—is a golden-hued, inert gas in an atomized form. It is a bronze mostly composed of aluminum as the principal alloying ingredient, supplemented by other metals such as iron. The inclusion of aluminum in these bronzes improves the corrosion resistance of coatings derived from these materials by facilitating the creation of a thin, durable surface oxide that serves as a protective layer for the copper-rich alloy. Other powder features include the following: nominal range: (−53 to +5) µm; manufacturing method—gas atomization; morphology—spheroidal; suggested process—Atmospheric Plasma Spray (APS).
- Metco 445 (Cu 9.5Al—nominal composition, chemical composition wt% nominal: 7.0–12.0Al organic binder—2.5) is a mechanically clad aluminum bronze that exhibits self-bonding during thermal spray processing as a result of a chemical reaction of the cladded components. Other powder features include the following: nominal range (−106 to +45) µm; manufacturing method—mechanically clad; morphology—spheroidal.

The printed samples (hereinafter named substrate) were coated utilizing Atmospheric Plasma Spray (APS) technology, specifically a SPRAYWIZARD-9MCE (Sultzer-Metco, Westbury, NY, USA) equipped with a USA/9 MB spraying gun. The parameters employed, contingent upon the utilized powder, were as follows: nitrogen and hydrogen pressure—3.4–3.7 bar/nitrogen gas flow—39–44 NLPM; hydrogen gas flow—6.6 NLPM; electric DC—400 A. The following were used for the powder dispenser: carrier gas flow—5.1–5.3 NLPM; air pressure—1.4 bar; material flow—130 g/min; spray distance—130 mm; number of passes—4.

The deposition rate of the microparticles remained constant. A laser pyrometer was employed throughout the operation to regulate the melting temperature of the samples.

The powder coatings were made on two substrates of PLA and HD PLA Green by using two composite cooper micropowders, as follows: PLA (substrate) covered with Metco 51F-NS, HD PLA (substrate) covered with Metco 51F-NS, PLA (substrate) covered withMetco 445, and HD PLA (substrate) covered with Metco 445.

The powder coatings were made in accordance with the established experimental plan, as shown in Table 1.

This paper aims to present only the results for experiments 2 and 4 for each substrate as these were the most representative.

The coated samples were characterized using the following equipment:

The X’Pert Pro MRD X-ray diffractometer (PANalytical, Almelo, The Netherlands) was used for X-ray diffraction analysis (XRD) with a voltage of 45 kV and a diffraction angle (2θ) that shifted from 10 to 90 degrees. The data were processed and the graphs were created using two X’Pert Data Collector programs: X’Pert High Score Plus version 3 and X’Pert Data Viewer version 2.2g (Malvern Panalytical, Malvern, UK).

A CETR UMT-2 microtribometer was utilized for micro-indentation, friction coefficient, and scratch tests. For scratch examination, a 0.4 mm NVIDIA blade was used, the samples were put on a table, and a 10 N vertical force was applied. The table was translated 10 mm in 60 s at 0.167 mm/s. The program recorded vertical force Fz, horizontal force Fx, duration, and horizontal distance Y (of the sample’s fixed mass) during the automatic test. The software determines the apparent friction coefficient (A-COF).

A Rockwell type indenter, with a 120° diamond tip cone and 200 µm peak radius, was used for the micro-indentation test. The samples were put on a table and subjected to a 10 N vertical force according to study methods and durations. Three samples of each experiment were collected for precise statistical calculations of hardness, Young’s modulus, and also for repeatability. The program logged the indenter’s vertical travel distance C, time, and vertical force Fz during the capacitive sensor automatic test. Process settings included a (0.2–20) N sensor, 30 s loading, 15 s holding, and 30 s unloading.

Roughness measurements of the coated surfaces were performed on the Zygo 3D ZeGage optical profilometer, MX software (6.2 version). Three measurements were performed for each coated sample.

In order to perform the differential scanning calorimetric analysis, four sample fragments (printed and coated) up to 5 mm in size, weighing less than 50 mg, were studied as follows: the differential scanning calorimeter available was NETZSCH type DSC 200 F3 Maya; sensitivity—1 W; thermal accuracy—0.1 K; and enthalpy accuracy—1%; equipment calibrated according to bismuth, indium, tin, and zinc standards; protective atmosphere—argon; test range—(20–200) °C; heated—10,000 rpm. The thermal analysis program used was the Proteus, offered by NETZSCH, tangent method. Also, the temperatures where half transformation (T50) occurred and the amount of heat dissipated and absorbed were measured.

The Mettler Toledo TGA/SDTA 851 (Columbus, OH, USA) equipment was used to calculate thermogravimetric curves (TG), derived thermogravimetric curves (DTG), and differential thermal analyses (DTA). The mass of the samples that were thermally decomposed was approximately 4.46 to 4.88 mg. It was operated at a flow rate of 20 cm^3^/min in air atmosphere. The study was performed at 25–700 °C with a heating rate of 10 °C/min. STAR^e^ SW 9.10 software produced by Mettler Toledo (Columbus, OH, USA) was used to process the thermogravimetric curves. The start (Tonset), peak (Tpeak), and end (Tend) temperatures of each thermal degradation phase were measured. In addition, the percentages of mass loss or residue (W%) were indicated.

A QUANTA 200 3D electron microscope (FEI Company, Fremont, CA, USA) was used to perform the SEM structural analysis.

## 3. Results and Discussion

### 3.1. DSC Analysis

**Figure 1:**
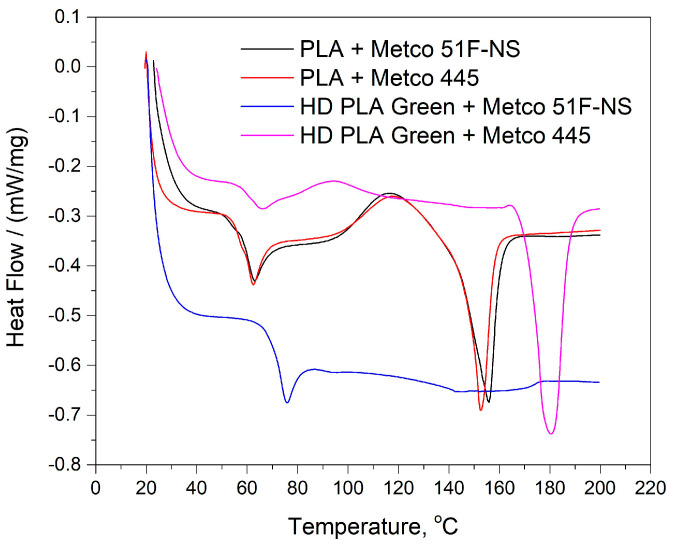
Thermal behavior of PLA and HD PLA Green substrates coated with Metco 51F-NS and Metco 445 copper-based micropowders.

Figure 1 shows the DSC curves for the four samples analyzed for the polylactic acid and HD PLA Green substrates; the curves are superimposed for both types of powders in order to better capture the variations that occur. These reflect the following specific transformations:

For the PLA substrate, the black and red curves reflect similar behaviors. The first transformation of PLA + Metco 51F-NS (black curve), on the initial part of the curve, was highlighted as an endothermic slope, associated with Tg (glass transition) at temperature 60.4 °C, and with a specific heat (required for the transformation) of 0. 320 J/(g*K).

The sample of PLA + Metco 445 (red curve) has Tg at 57.1 °C with Δ_Cp*_ = 0.524 J/(g*K), Table 2. This transformation was followed by a relaxation peak as well as a melting peak at 155.8 °C with heat absorbed of 19.34 J/g. In the case of the Metco 445 powder-coated sample, the melting point (endothermic peak) is 152.6 °C with 17.48 J/g of heat absorbed. The differences related to this transformation can be attributed to the table of the analyzed samples, but also to the powder layer covering the polylactic acid substrate.

Compared to the uncoated samples [62], the samples in the present study did not show exothermic peaks corresponding to the cold crystallization of the printed PLA material. Thus, it can be mentioned that the slow cooling of the material after testing did not highlight crystalline solidification of the polymer structure.

Following the behavior of the other substrate, HD PLA Green, it is observed that the sample coated with Metco 51F-NS (blue curve) shows glass transition at 71.5 °C with Δ_Cp*_ = 0.497 J/(g*K), followed by a relaxation peak, but it does not show a melting peak. It is worth mentioning that the test for this sample was repeated up to a temperature of 280 °C, but the melting of the sample was not recorded by the software. When opening the crucible, the sample was melted. According to the scientific literature [63], when thin wires are made of polymers, molecular orientations occur that influence the glass transition. Analogous to the behavior of semicrystalline polymers, the glass transition temperature is shifted to a somewhat higher temperature. Orientation (i.e., stretching) of semicrystalline polymers can lead to a significant increase in crystallinity. Thus, the lack of a melting point up to temperatures of 200 °C can be associated with reorientation of fibers/molecular chains in the substrate structure.

In the case of the HD PLA Green + Metco 445 sample (pink curve), the glass transition was highlighted at a temperature of 62.2 °C with a specific heat of 0.244 J/(g*K), as well as an endothermic minimum at a temperature of 180.5 °C associated with the melting point of the material, and the amount of absorbed heat being 31.71 J/g.

### 3.2. Thermogravimetric Analysis

**Figure 2:**
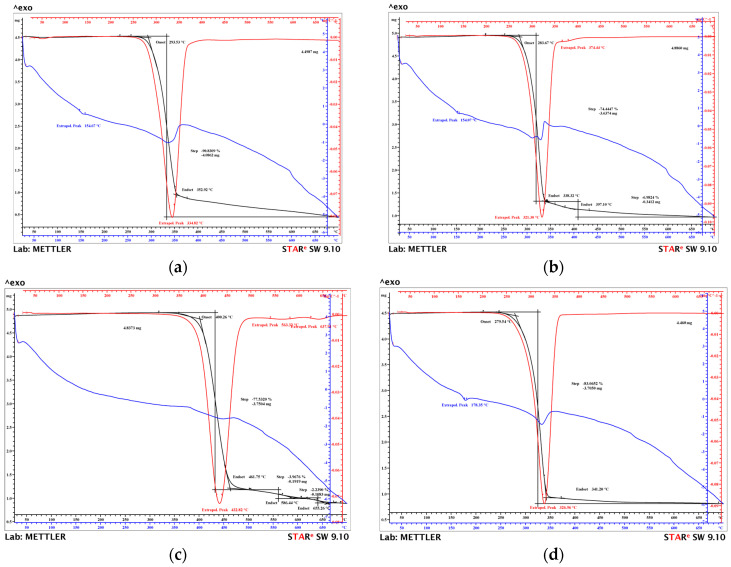
TG analysis of samples coated with copper-based composite powders: (**a**) PLA + Metco 51F-NS, (**b**) PLA + Metco 445, (**c**) HD PLA Green + 51F-NS, (**d**) HD PLA Green + Metco 445.

Understanding the thermogravimetric behavior of the samples coated with copper-based metal micropowder is essential for their use in applications that entail severe working conditions. This is due to the fact that whether wear resistance or thermal resistance occur, the study of thermogravimetric behavior is very important. It is desirable that the coated samples lead to improved mechanical and tribological characteristics but also maintain their thermal stability. The thermogravimetric (TG—in black), derivative thermogravimetric (DTG—in red), and differential thermal (DTA—in blue) curves for the four coated samples are shown in Figure 2.

The main thermogravimetric characteristics of the PLA + Metco 51F-NS, PLA + Metco 445, HD PLA Green + Metco 445, and HD PLA Green + Metco 445 samples are presented in Table 3.

Three of the samples coated with thin layers of copper-based metal particles, except for the HD PLA Green + Metco 51F-NS sample, show a major decomposition stage (Table 3), recorded around the temperature of (321 ÷ 335)°C, with a semi-significant mass loss of more than (74–90)%; this finding is attributed to the structural degradation of the basic constituent of the material, PLA. This stage consists of the formation of aromatic hydrocarbons, hydroxy-phenolic, and other compounds [64,65]. According to the manufacturer and the scientific literature [66,67,68], the biopolymer decomposes in considerable proportions in this temperature range and then completely, up to a temperature of 500 °C.

The PLA + Metco 445 sample also shows a degradation step at a temperature of about 375 °C, which occurs with a mass loss in a much lower percentage of 7%, a loss attributed to the thermal oxidation of the carbonic residue that arose from the PLA pyrolysis.

In the case of the HD PLA Green + 51F-NS sample, the major degradation step does not occur until a temperature of 432.87 °C, with a mass loss of 77.35%. This shift in the thermal degradation range, as explained in the DSC analysis, can be attributed to the coating that led to the reorientation of the fibers/molecular chains in the HD PLA Green biopolymer structure. Also, no exothermic transformation (visible in the case of the other samples) associated with the substrate melting point could be detected on the DTA curve (blue). On the DTG curve (red), two more degradation stages appear at 563.12 °C and 637.87 °C, with mass losses of about 6.2% that can be associated, as in the case of the other samples, to the thermal oxidation of the PLA carbonic residue, but also of other inorganic constituents of the polymer introduced by the manufacturer as binders [69].

At a temperature of 700 °C, a certain amount of residual mass is found, depending on the type of coating/substrate. It should be mentioned that the composite metal powders at the end of the analysis temperature have not yet reached the melting point of the component metals, copper (1083 °C), aluminum (660 °C), and iron (1538 °C), with their masses being found in the final obtained residue.

Figure 2 reflects the differential thermal curves (blue–blue) where the melting temperature of the substrates can be observed, which is very close to the values obtained by calorimetric analysis.

On the basis of the thermal analysis, the following conclusion can be drawn: calorimetric and thermogravimetric analysis indicate that, with the deposition of the metallic micro-layer on the copper base, the thermal stability of the material is not affected. In order to obtain an increase in this characteristic, more passes could be performed, but with an increase in the size of the metallic layer, it is possible that the substrate will not resist and will start to carbonize much earlier.

### 3.3. SEM Analysis

Since the chemical bond at the metal/polymer interface is very weak, the first step before coating was sample preparation by sandblasting and etching with a strong acid. For SEM analysis, small-sized samples (20 × 20 × 10) mm were obtained by mechanical cutting from the initially coated samples.

**Figure 3:**
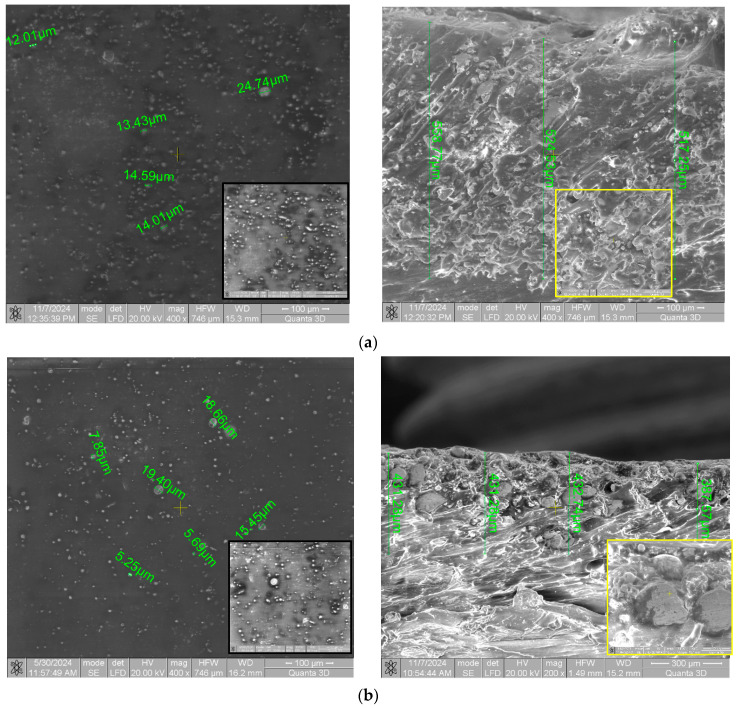

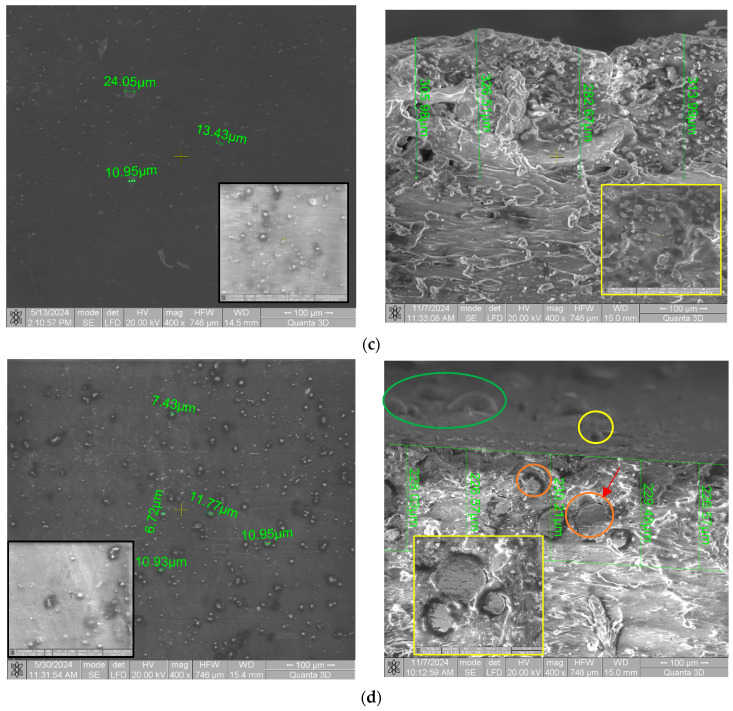
SEM cross-sectional analysis of samples coated with copper-based composite powder: (**a**) PLA + Metco 51F-NS, (**b**) PLA + Metco 445, (**c**) HD PLA Green + Metco 51F-NS, (**d**) HD PLA Green + Metco 445.

It is well known that the thermal conductivity of polymeric materials is one hundred times lower than that of metals, so that during deposition of a metallic layer on a polymeric layer, heat accumulates at the metal/polymer interface. Another important aspect is the plastic interaction between the metal particles and the substrate, because most of the impact energy on polymeric substrates is consumed in the deformation of the substrate, which leads to the embedding of the metal particles into the polymeric substrate, forming an initial state over which, during subsequent passes, new metal layers will be deposited. The subsequent impacts cause severe plastic deformation of the layer embedded in the polymeric mass, but mostly of the polymeric matrix, thus leading to the occurrence of erosions and to obtaining an uneven starting thickness over the entire surface of the coated sample. Most likely, for this very reason, in Figure 3, slight variations in the thickness of the deposited state can be observed for all four samples analyzed in the section (right images).

The sample with the polylactic acid substrate and coated with Metco 51F-NS micropowder highlighted a uniform deposition of metal particles on its surface (Figure 3a). The coating consists of constituent micro particles of the copper-based composite powder, having a size variation between 12–25 µm. Some of them retain their spherical shape due to very rapid cooling on contact with the biopolymer substrate. They do not flatten out in the form of splats, as is conventionally the case for coatings on metallic substrates [30]. The fact that the base matrix contains particles varying in shape and size in large quantity and uniform distribution leads to increased mechanical and tribological properties. In terms of deposited layer thickness, the highest state thickness obtained was 533.52 ± 22.17 µm because the PLA substrate was a soft one that allowed deeper penetration of the metal particles.

This behavior is also highlighted by the PLA substrate coated with Metco 445 (Figure 3b) where a state thickness of 420.72 ± 22.11 µm was obtained. The surface image of this sample shows a uniform deposition of microparticles, most of which are embedded, and polymeric mass.

The HD PLA Green substrate, being more impact-resistant than the PLA substrate, resulted in exhibiting less metal particle penetration. Thus, the HD PLA Green + Metco 51F-NS sample (Figure 3c) shows a deposited layer thickness of 307.29 ± 18.46 µm, while the Metco 445 coated sample (Figure 3d) has a much smaller layer thickness of 228.17 ± 1.66 µm. The surface images of both samples reflect a uniform deposition of (slightly embedded) particles with particle sizes between 6–25 µm.

From the cross-sectional images of the two samples with the HD PLA Green HD substrate, it can be seen that the metal particles in the initial layer were well embedded and adhered, but their impacts extruded the surrounding molten polymer, which remained as a film on top of them, acting as a separator between the constituent particles of the copper-based composite. This prevents their metallic bonding and the formation of a compact layer. In the upper part, instead, it is possible to observe that this polymer film acts as a binder of the particles.

Figure 3d shows slightly flattened particles on the surface of the sample (green mark) and inside the substrate due to the impact with the previously deposited metallic particles, which led to plastic deformations. Areas marked in an orange color indicate particles bordered by voids, which can be attributed to their dislocation during mechanical cutting. Also to be considered are the voids specific to 3D printing, the FDM method, where most probably have not completely disappeared due to the partial melting of the surface layer of the sample. The yellow-marked particle kept its initial spherical shape due to the contact with the cold polymeric layer.

### 3.4. XRD Analysis

The XRD investigations were mainly aimed at determining the structure of the PLA and HD PLA Green samples coated with composite bronze powders, Metco 51F-NS (Cu 9.5Al 1.2Fe) and Metco 445 (Cu 9.5Al), and at identifying the possible crystalline phases present.

**Figure 4:**
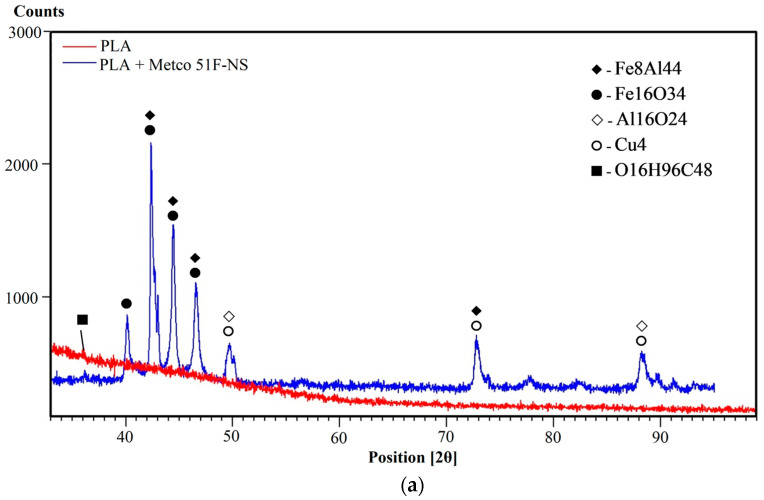

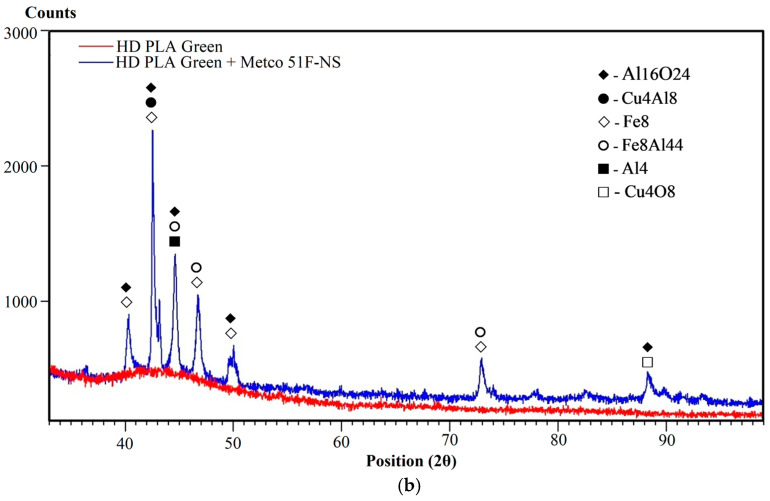
**XRD analysis** of printed samples coated with bronze composite powders: (**a**) PLA + Metco 51F-NS; (**b**) HD PLA Green + Metco 51F-NS.

Figure 4 shows the phase diffractograms for the two substrates (PLA and HD PLA Green) compared with the Metco 51F-NS state-coated samples, as follows: PLA—Metco 51F-NS, Figure 4a; and HD PLA Green—Metco 51F-NS, Figure 4b. The XRD spectra for the two substrates, PLA and HD PLA Green, reflect their semi-amorphous structures due to the broad peak shapes. However, they also show narrow maxima, which attests that crystallizations of certain substances such as polylactic acid (C_3_H_4_O_2_)_n_ occur, as reported in other studies [62,70]. Also, in the case of HD PLA Green, a peak symbolized by the square in Figure 4b is obtained at an angle of 35.836°, which, according to the scientific literature, corresponds to the compound O_16_H_96_C_48_ [71]. The O_16_H_96_C_48_ (racemic trans-1,2-cyclohexanediol) compound was probably used as building block (small complex structures) or chiral auxiliaries (natural molecule attached to reaction for orientation control) in order to easily achieve a desired outcome.

The diffractograms for the samples coated with the composite bronze powder, Metco 51F-NS, reflect a crystalline structure, emphasized by specific tall and thin peaks. The PLA + Metco 51F-NS sample (blue diffractogram—Figure 4a) shows a series of major peaks of different intensities, as follows: Fe_8_Al_44_ at angles of 42.358°, 44.400°, 46.816°, and 72.882° [72]; Fe_16_O_34_ at 2θ angles of 40.251°, 42.312°, 44.364°, and 46.878° [73]; Al_16_O_24_ (aluminum oxide—kappa) at angles of 40. 251°, 42.396°, 42.396°, 44.399°, and 47.485°, of lower intensity [74]; and Cu_4_: 50.419°, 74.078°, 89.874°, of lower intensity [75].

In the case of HD PLA Green PLA coated with Metco 51F-NS bronze powder (Figure 4b), crystallization peaks specific to the Al_16_O_24_ and Fe_8_Al_44_ compounds present on the PLA substrate also appear in this deposition, as expected. Other peaks with compounds similar to the previous sample also appear as follows: Cu_4_A_l8_ at an angle of 42.613° [76]; Fe_8_ for diffraction angles 40.301°, 42.590°, 46.766°, 50.809°, and 73.078° [77]; Al_4_ at a 2θ angle 44.693° [78]; and Cu_4_O_8_ for the low intensity angle of 88.555° [79].

**Figure 5:**
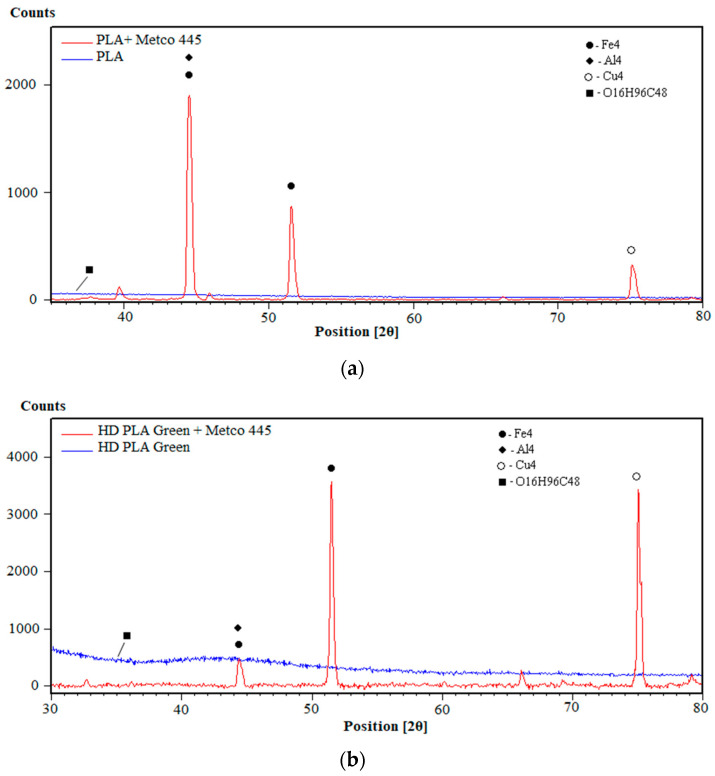
**XRD analysis** of printed samples coated with bronze composite powders: (**a**) PLA + Metco 445; (**b**) HD PLA Green + Metco 445.

In the case of XRD analysis for the PLA- and HD PLA Green-coated samples coated with a copper-based composite powder—Metco 445, Figure 5—the presence of the same compounds was observed at different intensities, as follows:

- The PLA substrate sample (Figure 5a) showed an intense peak at 2θ for the angle of 45.199° (Fe_4_) and 44.740° (Al_4_) [78]. Two other lower intensity peaks were associated with the presence of Cu_4_ at 74.926° [80], and Fe_4_ at 51.575° [78]. The lower intensity peaks from all these identified elements are part of the composition of the copper-based powder deposited on the substrate. The polymeric substrate as in the previous samples revealed the presence of the compound O_16_H_96_C_48_ [71], which was previously reported.
- The HD PLA Green HD substrate (Figure 5b) revealed two major peaks corresponding to Fe_4_ crystallization at 51.575°, 75.209 [78], and Cu_4_ at the same 2θ angle as the PLA substrate sample, 74.926° [80]. The peaks at 45.199° and 44.740° correspond to the present Al_4_ and Fe_4_ [78]. The compound O_16_H_96_C_48_ [71] is also found in this analyzed substrate.

### 3.5. Friction Coefficient Determination

The determination of the friction coefficient (COF) was realized by translational motions between a 6.35 mm diameter steel pin and the samples with the PLA substrate and HD PLA Green substrate. Tests were performed with a loading force of 10 N, linear translational motion velocity of 10 mm/s, over a distance of 10 mm, and 600 s testing time.

**Figure 6:**
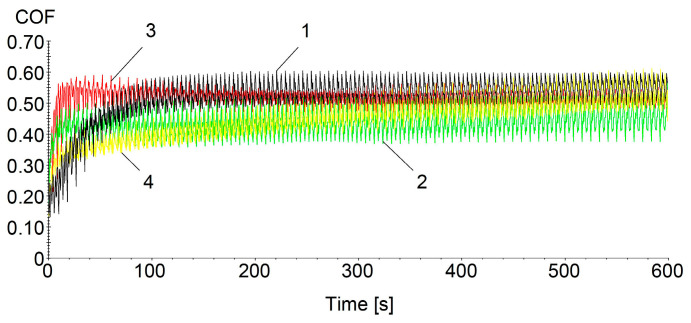
COF variation with test time for coated PLA and HD PLA Green, 1–4 the tested samples.

In Figure 6, the variation curves obtained when determining the friction coefficient for the samples proposed to be tested are as follows: curve 1 (black)—PLA coated with Metco 51F-NS; curve 2 (green)—PLA coated with Metco 445; curve 3 (red)—HD PLA Green coated with Metco 51F-NS; and curve 4 (yellow)—HD PLA Green coated with Metco 445. During the determinations, for all the materials studied, a progressive increase was observed in the first part of the test, about 20s (maximum COF value being reached, Table 4), after which the value of the coefficient stabilizes and remains the same until the end of the tests in the case of the PLA substrate-coated samples and HD PLA Green + Metco 51F-NS. The HD PLA Green + Metco 445 sample had different behavior, with the COF value increasing slightly until the end of the test when it reached the 0.665 maximum value. This was most likely due to the fact that the pin detached small coating particles from the sample surface, which were pushed to the edge of the test track, causing an increase in COF value throughout the test. The detachment of the micro particles may be attributed to a weaker adhesion of the Metco 445 composite powder on the HD PLA Green substrate. The highest mean value of the friction coefficient, 0.515 ± 0.06, was recorded by the HD PLA Green + Metco 51F-NS sample, followed by the HD PLA Green + Metco 445 sample (0.464 ± 0.08), Table 4.

Compared with the uncoated samples according to the literature [62], the COF value increased in the case of the copper-based composite powder coatings from 0.11 to about 0.430 in the case of the PLA coating, an increase of ≈290%. In the case of the HD PLA Green substrate, the increase is even higher, from 0.04 to about 0.490.

Thus, the deposition of the copper-based composite microparticles on the surface of the PLA and HD PLA Green substrates created a rough texture at the micrometer scale. This increases the effective contact area and hence the friction coefficient. In contrast to pure polymer surfaces, which can become slippery under load or high temperatures, the copper particle coating stabilizes friction over time, preventing rapid wear or friction decay.

### 3.6. Scratch Analysis

The scratch test was performed to determine the adhesion ability of the copper-based micropowder coatings on the surface of painted samples of biodegradable PLA and HD PLA Green.

The scratch analysis of the samples with the PLA substrate and coated with Metco 51F-NS (red curve) and Metco 445 (green curve) highlights the achievement of a high apparent coefficient of friction (A-COF) value for the Metco 445 copper-based composite powder coating. This is most likely caused by the higher particle size of the powder (compared to Metco 51F-NS) which, according to the manufacturer, is between 106 ÷ 45 µm.

**Figure 7:**
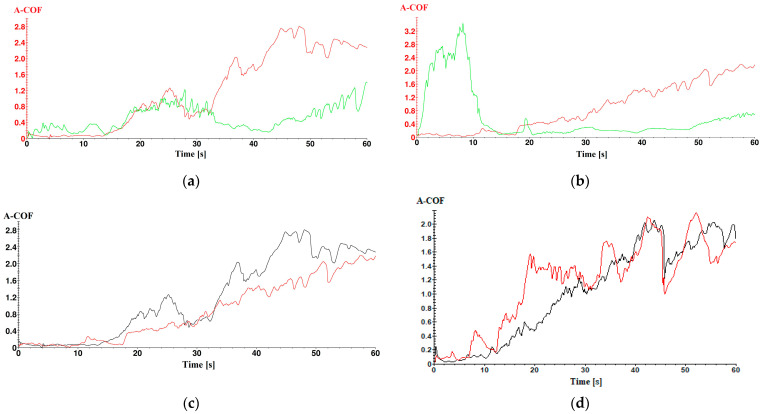
Variation in apparent A-COF with test time for samples coated with copper-based metal micropowder: (**a**) PLA substrate, coated with Metco 51F-NS—red curve; Metco 445—green curve. (**b**) HD PLA Green substrate, coated with Metco 51F-NS—red curve; Metco 445—green curve. (**c**) Metco 51F-NS coating: substrate PLA—black curve; substrate HD PLA Green—red curve. (**d**) Metco 445 coating: substrate PLA—black curve; substrate HD PLA Green—red curve.

For the sample with the PLA substrate (Figure 7a), an average A-COF value of 1.20 ± 0.95 is obtained, which is almost double that of the sample with the HD PLA Green substrate, which has A-COF value of 0.61 ± 0.79. This is most likely due to the mechanical characteristics of HD PLA Green HD PLA (such as impact strength 17.71 ± 0.32 kJ/m^2^) compared to PLA (15.87 ± 0.43 kJ/m^2^) [81]. Thus, the PLA substrate, being softer, allowed the microparticles to penetrate deeper than the samples with the HD PLA Green substrate, significantly influencing their adhesion to the polymeric substrate. It can also be observed that the HD PLA Green sample coated with Metco 445 (green curve) (Figure 7b) shows in the first 12 s of the test an A-COF maximum of 3.43; the explanation would be related to the deposition grain size. It is possible that the tip of the sharpener tool, also known as the pin, may have snagged a portion of the deposited material with a higher grain size.

The curves presented in Figure 7c,d show a sharp and gradual transition of A-COF, indicating that the strength of the adhesion and chemical bonds created between the deposited micro-layer and the polymeric substrate is good. The good adhesion of the layer is given by the penetration of the particles into the printed polymeric substrate, where the formation of a thin composite metal state (during the first passes) later leads to an even better adhesion of the metal particles deposited in the next waves.

Based on the curves in Figure 7c,d and Table 5, the scratch behavior of the samples during the 60 s of testing could be established; it can be observed that the A-COF value increases progressively in the type of testing for most of the samples, the maximum A-COF being reached in the last seconds of testing, except for the HD PLA Green sample coated with Metco 445, which is discussed above. This increase reflects the fact that the test pin detached metal microparticles from the sample surface, thus the sample gradually becomes rougher and rougher. Also, a large number of peaks occurring in the A-COF variation is observed, indicating good adhesion between the deposited layer and the polymeric material.

The highest mean value of the apparent coefficient of friction was recorded by the PLA sample coated with the Metco 51F-NS composite powder, 1.20 ± 0.95. The mean A-COF values are higher for the Metco 51F-NS powder for both substrates, indicating better adhesion to the two biodegradable substrates.

### 3.7. Surface Roughness Analysis

The highest mean value of Ra (surface roughness) was recorded by the PLA sample coated with the Metco 51F-NS composite powder, 3.15 ± 1.09 µm, indicating good adhesion of the powder on the PLA substrate (Table 6). The next value of roughness is also recorded by the PLA substrate, but this time coated with Metco 445, 1.93 ± 2.01 µm, revealing the higher permissiveness of polylactic acid in coatings due to its mechanical characteristics, and lower impact resistance than in the case of HD PLA Green. Regarding the standard deviation of the values obtained, the samples with the HD PLA Green substrate showed much lower values, indicating that the surface is smoother and the roughness does not vary as much as in the case of the PLA substrate.

The increased roughness of the polymeric surfaces coated with the copper-based composite powders leads to an increased effective contact area, which is useful in applications requiring high mechanical strength, and also favors the entrapment of lubricants in microscopic cavities, reducing friction and wear in controlled friction applications. Moreover, rough surfaces can facilitate bonding or interaction with other materials, being advantageous in subsequent lamination or coating processes.

### 3.8. Micro-Indentation Test

**Figure 8:**
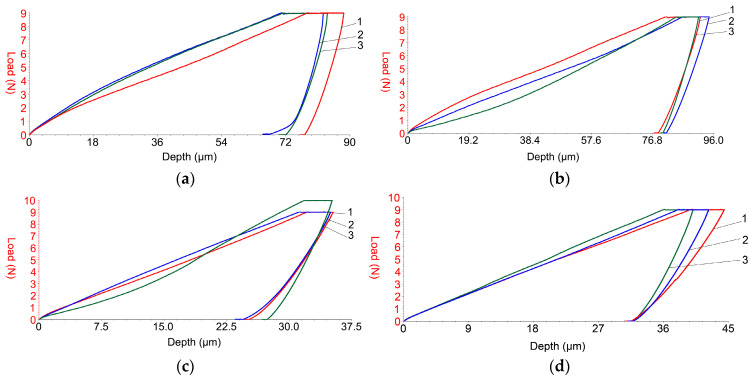
Results of micro-indentation tests for samples coated with copper-based metal micropowders: red curve—sample 1; blue curve—sample 2; green curve—sample 3. (**a**) PLA + Metco 51F-NS; (**b**) HD PLA Green + Metco 51F-NS; (**c**) PLA + Metco 445; (**d**) HD PLA Green + Metco 445.

Each type of metal powder deposited on the selected substrates (PLA and respective HD PLA Green) underwent three tests to achieve micro-indentation. Finally, repeated testing aimed to confirm the stability of the assay. Figure 8 shows the changes in pressing force as a function of penetration depth for each of the analyzed samples. The use of the UMT Test Viewer software package UMT Test Viewer (2.16) allowed the reading of both microhardness and Young’s modulus values. Table 7 contains the above-mentioned values. The samples coated with Metco 51F-NS exhibit much higher penetration depth values compared to the Metco 445 powder, regardless of the substrate type, which is probably due to the smaller particle size (−53 + 5) µm. The small particle size made it even easier for the particles to pass, during pressure spraying, through the top layer of the printed polymer (FDM method) characterized by voids and into the internal structure of the substrate, filling the voids characteristic of FDM printing, which was observed in the SEM analysis in the cross-section of the sample. The deposition of the Metco 445 powder resulted in a harder surface, almost 4 times harder than the other sample. This can be attributed to the size of the powder particles (−106 + 45 µm), which were uniformly arranged in the specific 3D printing spaces/voids; thus, the substrate surface acquired a much better surface quality and hardness than the initial, uncoated printed sample. Both Young’s modulus values (about 5.6 GPa) and microhardness values (0.25 GPa) are higher for the Metco 445 coatings (for both polymeric substrates), denoting that these surfaces are harder than those coated with Metco 51F-NS. This behavior is also justified by the chemical composition of the composite microcoatings.

The lowest dispersion of the results was obtained for the samples coated with Metco 445 (Figure 8), most likely due to the fact that the deposited layer was uniform. Also, the other tested samples did not show semi significant differences, as illustrated in Figure 8 and Table 7.

## 4. Conclusions

- The PLA + Metco 51F-NS and PLA + Metco 445 samples show similar thermal behaviors, but with notable differences in the values of the glass transition point (Tg) and melting points. The samples showed a relaxation peak followed by a melting point, which is higher for Metco 51F-NS (155.8 °C with 19.34 J/g absorbed) compared to Metco 445 (152.6 °C with 17.48 J/g absorbed). These differences are attributed to the interaction of the PLA substrate with the copper powder layers. The coated HD PLA Green substrates exhibit distinct behaviors depending on the powder used. Metco 51F-NS leads to a molecular reorientation that eliminates the melting point in the analyzed temperature range, while Metco 445 allows for the identification of a clear melting point at a significantly higher temperature. These differences emphasize the influence of powders on the thermal properties of substrates.
- The calorimetric and thermogravimetric analyses indicate that the deposition of the thin layer of copper-based metal micropowder does not affect the thermal stability of the material. Most of the coated samples show a main degradation stage between 321 °C and 335 °C, associated with significant mass loss (74–90%), due to the structural degradation of PLA/HD PLA Green. At 700 °C, a residual mass remains, which includes metal components that have not reached their melting points (e.g., copper, aluminum, iron).
- The PLA substrate is more suitable for thicker and better integrated layers due to its softness and its ability to allow deep penetration of metal particles. The HD PLA Green substrate, being stiffer, provides thinner and more impact-resistant layers, but limits compact metal bonds due to the polymer film formed at the interface. The substrate choice depends on the desired application, balancing layer thickness with structural strength and uniformity.
- Depositions of copper-based composite powders (Metco 51F-NS and Metco 445) generate similar crystalline structures on the PLA and HD PLA Green substrates, but the intensity and nature of crystallization varies with the substrate. The PLA substrate, being softer, favors a more intense crystallization, whereas the stiffer HD PLA Green substrate causes variations in crystalline intensity and distribution, suggesting a different interaction between the deposited layer and the substrate.
- PLA and HD PLA Green show different behaviors in the scratch test due to their mechanical properties. PLA, being softer, allows for a higher adhesion of metal particles, while HD PLA Green, being stiffer, resists penetration more, but maintains a good uniformity of the deposited layer. In both cases, the Metco 445 powder, due to its larger particle size, tends to give higher A-COF values, suggesting quality adhesion.
- The coating of the PLA and HD PLA Green materials with copper particles has increased the efficiency of the coefficient of friction by improving surface texture, stabilizing mechanical interaction, and reducing slip, providing superior and reliable tribological performance in industrial applications.
- The PLA substrate demonstrates a higher permittivity for adhesion of coatings, indicated by high mean roughness values, but with significant variations (large standard deviation), reflecting a less uniform surface.
- The PLA and HD PLA Green substrates show similar behaviors in micro-indentation tests, but differences in mechanical properties influence the lamination results. Metco 445 generates harder and stiffer surfaces due to its larger particles and more uniform distribution and is more effective for achieving a tough surface. In contrast, Metco 51F-NS provides deeper penetration and filling of internal substrate spaces but results in softer surfaces. HD PLA Green provides a more stable and penetration-resistant base than PLA, but both substrates benefit significantly from the improved properties of the metal coating.

The results indicate that the samples exhibited significant chemical bonding at the interface between the metallic layers and the bio-based substrates. Consequently, these coated materials are suitable for particular industrial applications (plane surfaces/simple geometry) necessitating elevated surface hardness and thermal resistance. They can effectively substitute numerous non-biodegradable polymeric materials utilized in diverse applications, including those within the automotive, electronics, and medical sectors (e.g., disposable cutlery, furniture surfaces, utensil handles, etc.).

## Figures and Tables

**Table 1:** Experimental plan.

Exp. No.	Coating (Powder)	Substrate	Number of Passes	Distance (mm)
1	Metco 51F-NS	PLA	3	130
		HD PLA Green		
2	Metco 51F-NS	PLA	4	130
		HD PLA Green		
3	Metco 51F-NS	PLA	3	130
		HD PLA Green		
4	Metco 445	PLA	4	130
		HD PLA Green		
5	Metco 445	PLA	3	130
		HD PLA Green		
6	Metco 445	PLA	4	130
		HD PLA Green		

**Table 2:** Phase transformations of samples coated with copper-based metal micropowders.

Sample	Glass Transition [°C]				Melting Point [°C]	
	T_start_	T_peak_	T_end_	Δ_Cp*_ [J/(g*K)]	T_peak_	ΔH/m [J/g]
PLA + Metco 51F-NS	58.5	60.4	61.4	0.320	155.8	19.34
PLA + Metco 445	57.0	57.1	60.1	0.524	152.6	17.48
HD PLA Green + Metco 51F-NS	70.2	71.5	73.2	0.497	-	-
HD PLA Green + Metco 445	57.1	62.2	64.7	0.244	180.5	31.71

Critical transformation temperatures. T_start_: starting temperature; T_peak_: average temperature; T_end_: end temperature of transformation (determined using the tangent method), ΔH/m: amount of heat dissipated/absorbed (using rectilinear basis).

**Table 3:** Thermogravimetric characteristics of samples coated with copper-based metal micropowders.

Sample	T_start_ [°C]	T_peak_ [°C]	T_end_ [°C]	Ma [%]	DTA Characteristic	Reziduu [mg]
PLA + Metco 51F-NS	293.82	334.60	352.83	90.80	exo	4.08
PLA + Metco 445	283.60	321.39	338.25	74.40	exo	3.64
HD PLA Green + Metco 51F-NS	400.27	432.87	461.70	77.35	exo	3.74
HD PLA Green + Metco 445	279.40	326.99	341.34	83	exo	3.70

T_start_, temperature at which thermal degradation begins at each stage; T_end_, temperature at which thermal degradation ends at each stage; T_peak_, temperature at which rate of degradation at each stage is maximum; Ma %, percent mass loss at each stage; residue, amount of degraded sample remaining at temperature above 700 °C.

**Table 4:** Average COF values for tested samples.

Sample	COF Average Value	Max COF
PLA + Metco 51F-NS	0.416 ± 0.07	0.556
PLA + Metco 445	0.449 ± 0.07	0.656
HD PLA Green + Metco 51F-NS	0.515 ± 0.06	0.652
HD PLA Green + Metco 445	0.464 ± 0.08	0.665

**Table 5:** Results of tribological testing of samples coated with copper-based composite micropowder.

Coating (Powder)	Substrate	A-COF Medium	A-COF Maximum	Time of A-COF Maximum [s]	Ff (A-COF Max) [N]
Metco 51F-NS	PLA	1.20 ± 0.95	2.8	47.93	22.05
	HD PLA Green	0.87 ± 0.70	2.19	57.55	21
Metco 445	PLA	0.51 ± 0.29	1.41	59.94	14.02
	HD PLA Green	0.61 ± 0.79	3.43	8.12	8.63

**Table 6:** Results of surface roughness analysis.

Sample	Slice	*Ra* (µm)	Sample	Slice	*Ra* (µm)
PLA + Metco 51F-NS	1	4.36	HD PLA Green + Metco 51F-NS	1	0.76
	2	2.24		2	0.58
	3	2.86		3	0.51
*Average*	-	*3.15 ± 1.09*	*Average*		*0.62 ± 0.13*
PLA + Metco 445	1	4.25	HD PLA Green + Metco 445	1	0.271
	2	0.72		2	1.321
	3	0.86		3	0.420
*Average*	-	*1.93 ± 2.01*	*Average*		*0.671 ± 0.568*

where *Ra* is the arithmetic average of the absolute values of the profile height deviations from the centerline.

**Table 7:** Results obtained from micro-indentation test of printed samples coated with copper-based metal micropowders.

Material	Sample No.	Maximum Load (N)	Maximum Depth (µm)	Young’s Modulus (GPa)	Microhardness (GPa)
PLA + Metco 51F-NS	1	8.937	78.460	2.992	0.0881
	2	8.891	82.514	3.663	0.0793
	3	8.917	74.391	3.209	0.0949
	*Average*	*8.915 ± 0.023*	*78.455 ± 4.061*	*3.288 ± 0.342*	*0.0874 ± 0.007*
HD PLA Green + Metco 51F-NS	1	8.940	91.746	1.948	0.0719
	2	8.963	86.654	2.157	0.0783
	3	8.936	98.816	1.955	0.0632
	*Average*	*8.946 ± 0.014*	*92.405 ± 6.108*	*2.02 ± 0.118*	*0.0711 ± 0.007*
PLA + Metco 445	1	8.959	35.261	5.429	0.2568
	2	8.997	33.572	6.371	0.2666
	3	8.979	41.261	5.133	0.2119
	*Average*	*8.978 ± 0.019*	*36.698 ± 4.041*	*5.644 ± 0.646*	*0.2451 ± 0.029*
HD PLA Green + Metco 445	1	8.997	31.603	5.749	0.2926
	2	8.976	35.230	6.171	0.2518
	3	8.979	41.261	5.133	0.2119
	*Average*	*8.984 ± 0.011*	*36.031 ± 4.878*	*5.684 ± 0.522*	*0.252 ± 0.04*

## Data Availability

The original contributions presented in this study are included in the article. Further inquiries can be directed to the corresponding authors.
